# Body composition, sarcopenia, and frailty among Northern seasonal migrant older adults: a cross-sectional study

**DOI:** 10.1186/s12889-026-28960-3

**Published:** 2026-08-08

**Authors:** Bing Liang, Xiaoning Sun, Beibei Wang, Ting Yang, Jiaojiao Xing, Qing Xu

**Affiliations:** 1 Nutrition Department, Hainan Hospital of P.L.A General Hospital, Sanya, 572000 China; 2 Healthcare Department, Hainan Hospital of P.L.A General Hospital, Sanya, 572000 China; 3 Center Laboratory, Hainan Hospital of P.L.A General Hospital, Sanya, 572000 China; 4 Department of Nutrition, The First Medical Center of the Chinese P.L.A General Hospital, Beijing, 100853 China

**Keywords:** Migrant elders, Body composition, Sarcopenia, Visceral obesity, Frailty, Nutritional status, Bioelectrical impedance analysis

## Abstract

**Background:**

To provide preliminary data on body composition, muscle health, nutritional status, and frailty among seasonal migrant older adults who overwinter in Hainan, and to identify potential health risks in this growing mobile aging population.

**Methods:**

A cross-sectional study was conducted among 86 northern migrant elders recruited at Hainan Hospital of the PLA General Hospital between October 2023 and January 2024. Assessments included bioelectrical impedance analysis (BIA), handgrip strength, calf circumference, gait speed, Mini Nutritional Assessment-Short Form (MNA-SF), and frailty evaluation using the FRAIL scale. Adjusted comparisons and multivariable logistic regression were conducted to examine associations.

**Results:**

No participant met the diagnostic criteria for sarcopenia. Despite regular physical activity and mostly normal nutritional status, excessive adiposity was prevalent: 74.4% exceeded body fat standards and 68.6% had visceral obesity. Early muscle weakness was evident, with 13.9% showing lower grip strength (adjusted *P* < 0.001). Participants at nutritional risk had significantly lower body mass index (BMI) (adjusted *P* = 0.0005), fat mass (adjusted *P* = 0.0040), visceral fat area (adjusted *P* = 0.0284), and appendicular skeletal muscle mass index (ASMI) (adjusted *P* = 0.0007). Prefrail individuals exhibited lower grip strength (adjusted *P* = 0.0116) and lower gait speed (*P* < 0.001). Overall, risks of visceral obesity, early sarcopenia features, and prefrailty coexisted despite the absence of diagnosed sarcopenia.

**Conclusions:**

Seasonal migrant older adults show active lifestyles yet significant hidden vulnerabilities. Strategies including routine screening, resistance training, and nutritional optimization may valuable in future preventive interventions, though further evaluation is required.

**Clinical trial number:**

ChiCTR2300073205.

**Supplementary Information:**

The online version contains supplementary material available at https://doi.org/10.1186/s12889-026-28960-3.

## Background

Population aging has accelerated rapidly in recent decades, with China entering a stage of deep aging earlier than many other countries. Nationwide statistics show that individuals aged 65 years and above accounted for more than 14% of the population in 2021, and this proportion will continue to increase. In China, the older adult population is projected to exceed 400 million around 2035, accounting for approximately 30% of the total demographic [1–3]. Against this demographic backdrop, a unique lifestyle pattern, seasonal cross-regional migration among older adults, has emerged. These “seasonal migrant older adults” typically relocate from colder northern regions to warmer southern regions, such as Hainan, during the winter months [4]. This lifestyle pattern closely parallels the “snowbird” phenomenon in Western countries, situating this cohort within a broader global public health context [5]. While previous studies have extensively explored frailty and sarcopenia in general community-dwelling older adults, seasonal migrants face distinct changes in their environment, daily habits, and overall quality of life during relocation [6]. Hainan’s favorable climate and environment have made it a major destination, attracting hundreds of thousands of elderly migrants annually. This rapidly growing group represents a novel aging population with distinctive mobility, lifestyle behaviors, and health-care needs [7].

Body composition-related health conditions, including frailty, sarcopenia, and nutritional risk, are highly prevalent among older adults and strongly associated with functional decline, disability, hospitalization, and mortality [8, 9]. Frailty reflects decreased physiological resilience; sarcopenia involves age-related loss of muscle mass and strength; and nutritional risk often coexists with both conditions, forming a complex interplay that exacerbates adverse outcomes [10]. While these conditions are common in all older populations, migrant elders may face unique challenges. Seasonal relocation can lead to changes in dietary patterns and difficulties in accessing medical care in a new location, changes in physical activity patterns, and potential instability in social support [11]. These factors may affect body composition, muscle health, and overall functional capacity, making migrant elders potentially more vulnerable, or heterogeneously vulnerable, compared with community-dwelling non-migrant peers [12]. Consequently, their physiological vulnerability may differ significantly from static non-migrant populations during seasonal relocation.

Although numerous studies have examined frailty, sarcopenia, and nutritional status in general older adult populations, research focusing specifically on migrant elders remains scarce [13, 14]. Existing evidence from China is limited, often based on small local samples, and rarely incorporates comprehensive assessments of body composition, muscle function, and frailty using standardized tools [15]. Moreover, data on northern migrant elders who overwinter in Hainan, a population that has expanded significantly in recent years, are particularly insufficient [6]. As a result, the basic health profile of this group remains unclear, and the associated risks and clinical implications are not well understood.

To address this gap, the present study aimed to conduct a cross-sectional survey to depict the baseline characteristics of body composition, muscle function, and frailty status among northern migrant elders who overwinter in Hainan. The goal is to provide preliminary data for understanding the health status of this unique population, identifying potential health risks, and laying the foundation for future longitudinal studies and targeted intervention strategies.

## Methods

### Study design and participants

This study adopted a cross-sectional design and was conducted at Hainan Hospital of the People’s Liberation Army General Hospital between October 2023 and January 2024. A total of 86 migrant elders originating from northern China, including Heilongjiang, Jilin, Liaoning, Beijing, Tianjin, Hebei, and northeastern Inner Mongolia, were recruited. Eligible participants were aged ≥ 60 years, resided in Hainan for more than three months during seasonal migration, had basic self-care ability, and were able to stand independently. All participants were required to read, comprehend, and communicate effectively, be fully informed of the study purpose, and to voluntarily consent to participation and completion of all study procedures. Exclusion criteria included malignant tumors, hematological diseases, severe organic disorder, psychiatric illnesses, dementia or mild cognitive impairment, anticipated surgery within one year, or any acute or infectious disease occurring within one month before participation. Recruitment was conducted through voluntary enrollment after public announcements via the hospital’s official WeChat public account. This study employed a cross-sectional design, and the sample size was calculated using the estimation method for an overall proportion in cross-sectional studies. Based on the reported combined prevalence of frailty and pre-frailty among community-dwelling older adults in China (approximately 60%) [16], with a two-sided significance level of α = 0.05 ($\:{Z}_{1-\alpha\:/2}=1.96$), and an allowable error set at 0.2 times the estimated population proportion (d = 0.12), the minimum required sample size was calculated to be 64 participants. Considering the possibility of missing data and invalid measurements, a 20% attrition rate was allowed for, resulting in a final required sample size of 80 participants. A total of 86 participants were ultimately included, indicating that the sample size was adequate to meet statistical requirements.

The study was approved by the Medical Ethics Committee of Hainan Hospital, Chinese PLA General Hospital (Approval No. S2022-48), all participants were informed about the study protocol and provided written informed consent to participate. This cross-sectional study utilized a portion of the baseline data from a larger prospectively registered observational cohort study (Clinical Trial Registration: ChiCTR2300073205).

### Data collection

A structured questionnaire was used to collect information on participants’ basic demographic characteristics, place of origin, lifestyle habits, and patterns of physical activity.

Body composition assessment was analyzed using bioelectrical impedance analysis (BIA) [17]. BIA was selected because it is radiation-free, noninvasive, and rapid, maximizing acceptability among older adults, and due to the unavailability of dual-energy X-ray absorptiometry (DXA) facilities in the study setting. To maximize data accuracy, all measurements were performed by the same trained researcher. Assessments were conducted in the morning after an overnight fast and after bladder emptying. Alcohol consumption was prohibited within 24 h before measurement. Participants were asked to remove metal objects and wear light clothing before the assessment. The X-SCAN PLUS II analyzer (Jawon Medical, Korea) was used to evaluate body composition and estimates “body age” based on sex and biological age. Measurements included body weight, body mass index (BMI), body fat percentage, fat-free mass, visceral fat area, intracellular water, extracellular water, muscle mass, appendicular skeletal muscle mass index (ASMI), and phase angle (PA), which serves as an indicator of cellular membrane integrity and overall nutritional status [18]. The device uses multi-segmental and multi-frequency measurements (1, 5, 20, 250, 500, and 1000 kHz) to simultaneously obtain resistance and reactance values [19]. Body fat percentage classifications (normal range: 15%–20% for men and 20%–30% for women) were based on criteria from the X-SCAN plus II analyzer, with “excessive body fat percentage” defined as exceeding these thresholds (> 20% for men, > 30% for women). High BMI and “above-standard BMI” were evaluated according to the age-specific normal BMI ranges recommended in the Chinese Dietary Guidelines (2022 edition): 18.5–23.9 kg/m² for ages 60–64, 20.0–26.9 kg/m² for ages 65–79, and 22.0–26.9 kg/m^2^ for ages ≥ 80, with values above these ranges considered above-standard. Other classifications were based on proprietary device-generated categories from the analyzer: “visceral obesity” was defined as a visceral fat area ≥ 100 cm^2^, “well-developed muscle” evaluations were based on the standard score and reference range generated by the device (70.5 [55.8–59.4]), and “balanced type” was defined via the manufacturer’s built-in muscle-fat symmetry matrix. Regarding device outputs, PA represents a direct biophysical measurement derived from raw resistance and reactance at specific frequencies, whereas “body age” is a proprietary algorithm-derived estimate calculated based on biological age, sex, and fat/muscle parameters. Furthermore, physical activity patterns were assessed via self-report focusing on activities during their previous 3 months of residence in Hainan.

Sarcopenia was assessed following the 2019 Asian Working Group for Sarcopenia (AWGS) diagnostic criteria and the 2024 Chinese guideline for sarcopenia management. The assessment included evaluation of muscle strength, physical performance, and skeletal muscle mass. Muscle strength was measured using handgrip strength, with thresholds defined as < 28 kg for men and < 18 kg for women, indicating low muscle strength. Physical performance was evaluated using the five-time chair stand test and usual gait speed over 6 m; a chair stand time > 12 s or a gait speed < 1 m/s was considered low physical performance. According to the guidelines, the exact cut-off values for calf circumference indicating low muscle mass indicators were set at < 34 cm for men and < 33 cm for women. Skeletal muscle mass was measured using BIA to calculate the skeletal muscle mass index (SMI), with cutoffs of < 7.0 kg/m² in men and < 5.7 kg/m² in women indicating low muscle mass. Diagnostic classification was as follows: low muscle strength or low physical performance with normal muscle mass indicated possible sarcopenia; the presence of low muscle mass combined with either low muscle strength or low physical performance defined sarcopenia; and the combination of low muscle mass, low muscle strength, and low physical performance was classified as severe sarcopenia. These diagnostic standards were applied uniformly to ensure accurate identification and classification of sarcopenia in the study population [20–22].

Nutritional status was evaluated using the Mini Nutritional Assessment–Short Form (MNA-SF), with scoring categories of 12–14 points, normal nutritional status; 8–11 points, at risk of malnutrition; 0–7 points, malnutrition [23]. Frailty was initially evaluated using both the Fried Frailty Phenotype scale and the FRAIL scale. However, for statistical analysis and group classification in this study, the FRAIL scale was utilized. The FRAIL scale is a 5-item questionnaire assessing Fatigue, Resistance (difficulty walking up 10 steps alone without resting), Ambulation (difficulty walking several hundred yards alone), Illnesses (5 or more specified illnesses), and Loss of weight (> 5% in the past year). Each item is scored 0 or 1. Total scores range from 0 to 5, with participants explicitly categorized into three standard frailty categories: robust (score 0), prefrail (score 1–2), and frail (score 3–5) [24]. In this context, “slowness” was operationalized via the self-reported Ambulation item rather than an objective gait speed cut-off. Objective gait speed was measured separately as part of the physical performance assessment.

### Statistical analysis

All statistical analyses were performed using SPSS version 27.0 (IBM Corp., Armonk, NY, USA). Continuous variables with normal distribution were expressed as mean ± standard deviation (mean ± SD), whereas those with skew distribution were expressed as medians with interquartile ranges. Categorical variables were presented as frequencies and percentages. Comparisons between groups were conducted using the chi-square test for categorical variables and one-way analysis of variance (ANOVA) or nonparametric tests for continuous variables, as appropriate. To adjust for potential confounding variables, comparisons of body composition and functional indicators across groups (i.e., between patients with normal muscle strength and those with possible sarcopenia, between patients with normal nutritional status and those at risk of malnutrition, and between robust and prefrail participants) were adjusted for age and sex using analysis of covariance (ANCOVA). P-values were further corrected for multiple comparisons using the Holm-Bonferroni method. In addition, an exploratory multivariable logistic regression model was used to examine factors associated with frailty, including age, sex, and variables that showed significant differences between the robust and prefrail groups. In the logistic regression model, gait speed was rescaled in 0.1 m/s increments for interpretation. The results of exploratory multivariable logistic regression were shown in odds ratio (OR) with 95% confidence interval (CI). A P-value < 0.05 was considered statistically significant.

## Results

### Demographic characteristic

Among the 86 participants, 39 (45.35%) were men and 47 (54.65%) were women. Most were Han ethnicity (95.35%). The majority were aged 65–79 years (74.42%), followed by 60–64 years (19.77%) and ≥ 80 years (5.81%). Participants came from eight northern regions, mainly Liaoning (32.56%), Beijing (24.42%), and Heilongjiang (19.77%). Overall, 51.16% reported a family history of chronic disease, and 65.88% had a medical history. Most were nonsmokers (83.72%) and nondrinkers (82.56%). Walking was the predominant exercise type (84.88%), with smaller proportions engaging in swimming, calisthenics, dancing, Tai Chi, table tennis, yoga, resistance training, jogging, cycling, or Qigong. The mean daily exercise duration was 1.33 ± 0.64 h. The mean duration of seasonal residence was 4.91 ± 2.43 years. All participants originally lived in urban areas. Regarding socioeconomic characteristics, all participants reported a monthly income between 3001 and 5000 RMB, and 100% had social security coverage. Regarding chronological versus body age, 54.7% had a chronological age greater than their body age, 16.3% had equal ages, and 29.1% had a chronological age lower than their body age (Table 1).

**Table 1:** Baseline characteristics of participants

Variable	Category	Value (*N* = 86)
Sex [*n* (%)]	Male	39 (45.35%)
	Female	47 (54.65%)
Age (years) [*n* (%)]	60–64	17 (19.77%)
	65–79	64 (74.42%)
	≥ 80	5 (5.81%)
Ethnicity [*n* (%)]	Han	82 (95.35%)
	Minority	4 (4.65%)
Province of origin [*n* (%)]	Liaoning	28 (32.56%)
	Beijing	21 (24.42%)
	Heilongjiang	17 (19.77%)
	Jilin	7 (8.14%)
	Shandong	4 (4.65%)
	Shanxi	4 (4.65%)
	Inner Mongolia	3 (3.49%)
	Hebei	2 (2.33%)
Education level [*n* (%)]	Bachelor’s degree or above	31 (36.05%)
	Associate degree	19 (22.09%)
	Vocational school	15 (17.44%)
	High school	11 (12.79%)
	Middle school or below	10 (11.63%)
Monthly income [*n* (%)]	< 3000 RMB	2(2.33%)
	3001–5000 RMB	24(27.91%)
	> 5000 RMB	60(69.77%)
Commercial insurance [*n* (%)]	Yes	28 (32.56%)
	No	58 (67.44%)
Social security [*n* (%)]	Yes	86 (100.00%)
	No	0 (0.00%)
Own housing in Hainan [*n* (%)]	Yes	42 (48.84%)
	No	44 (51.16%)
Regular medical checkup [*n* (%)]	Yes	78 (90.70%)
	No	8 (9.30%)
Perceived medical cost in Sanya [*n* (%)]	High	21 (24.42%)
	Reasonable	65 (75.58%)
Reasons for seasonal migration [*n* (%)]*	Air quality	70 (81.40%)
	Temperature	69 (80.23%)
	Sunlight	39 (45.35%)
	Diet	24 (27.91%)
Perceived health improvements [*n* (%)]*	Rheumatism	1 (1.16%)
	Joints	3 (3.49%)
	Respiratory function	41 (47.67%)
	Mobility/Physical activity	28 (32.56%)
	Immunity	3 (3.49%)
	Skin	1 (1.16%)
	Sleep quality	31 (36.05%)
	Digestive function	7 (8.14%)
	Mood status	35 (40.70%)
	Blood pressure	32 (37.21%)
	No obvious change	6 (6.98%)
Duration of seasonal migration (years)	Mean ± SD	4.91 ± 2.43
	Median (Q1, Q3)	4 (3, 6)
	Min, Max	2, 12
Family History [*n* (%)]	Yes	44 (51.16%)
	No	42 (48.84%)
Medical History [*n* (%)]	Yes	56 (65.88%)
	No	29 (34.12%)
Smoking [*n* (%)]	Yes	14 (16.28%)
	No	72 (83.72%)
Alcohol Consumption [*n* (%)]	Yes	15 (17.44%)
	No	71 (82.56%)
Exercise Type [*n* (%)]*	Table Tennis	4 (4.65%)
	Resistance Training	2 (2.33%)
	Jogging	2 (2.33%)
	Cycling	2 (2.33%)
	Qigong	1 (1.16%)
	Tai Chi	7 (8.14%)
	Dancing	8 (9.30%)
	Swimming	11 (12.79%)
	Yoga	2 (2.33%)
	Walking	73 (84.88%)
	Calisthenics	12 (13.95%)
	None	1 (1.16%)
Daily Exercise Duration (h/day)	Mean ± SD	1.33 ± 0.64
Weekly Exercise Duration (h/week)	Mean ± SD	9.19 ± 4.27
Chronological vs. body age [*n* (%)]	Chronological age > body age	47 (54.70%)
	Chronological age = body age	14 (16.30%)
	Chronological age < body age	25 (29.10%)

*Participants could select multiple activities or categories

### Body composition

Among all participants, 74.42% had an excessive body fat percentage, including 76.92% of men (> 20%) and 72.34% of women (> 30%). A total of 73.26% were classified as above-standard BMI, while 8.14% were underweight. Visceral adiposity was common: 67.44% had visceral obesity and 1.16% had severe visceral obesity, whereas only 3.49% were predominantly subcutaneous. Low muscle mass was observed in 33.33% of men and 25.53% of women. Muscle mass and fat-free mass were significantly higher in men, and nearly half of men (48.72%) fell into the “well-developed” muscle category, compared with only 4.26% of women. Both PA and ASMI were within normal ranges overall, but men showed significantly higher values than women (*P* < 0.001 for both). Age-related patterns showed that body fat percentage, fat mass, and visceral fat area increased with age. Regarding age-related variations, participants aged 65–79 years exhibited a significantly lower basal metabolic rate compared with those aged 60–64 years (*P* = 0.020). However, differences in PA, ASMI, and fat-free mass across age categories were not statistically significant (all *P* > 0.05). Characteristics of body composition parameters are presented in Table 2 and Table S1.

**Table 2:** Characteristics of body composition analysis of the migrant elders with different genders

	Overall (*n* = 86)	Male (*n* = 39)	Female (*n* = 47)	*P*
Age (years) [median (range)]	70.00 (65.25-74.00)	69.00 (65.00-74.50)	70.00 (66.00–73.00)	0.725
Age category [*n* (%)]				0.581
60–64	17 (19.77%)	9 (23.08%)	8 (17.02%)	
65–79	64 (74.42%)	27 (69.23%)	37 (78.72%)	
≥ 80	5 (5.81%)	3 (7.69%)	2 (4.26%)	
BMI (kg/m^2^) [mean ± SD]	24.05 ± 2.99	24.33 ± 3.05	23.82 ± 2.96	0.438
BMI category [*n* (%)]				0.977
Below standard	7 (8.14%)	3 (7.69%)	4 (8.51%)	
Normal	16 (18.60%)	7 (17.95%)	9 (19.15%)	
Above standard	63 (73.26%)	29 (74.36%)	34 (72.34%)	
Body fat percentage (%) [median (range)]	29.00 (24.22–32.18)	24.20 (20.25–26.35)	32.00 (29.70–35.10)	< 0.001
Body fat percentage category [*n* (%)]				0.813
< Standard	22 (25.58%)	9 (23.08%)	13 (27.66%)	
≥ Standard	64 (74.42%)	30 (76.92%)	34 (72.34%)	
Fat mass (kg) [median (range)]	18.35 ± 5.42	16.91 ± 6.02	19.55 ± 4.60	0.024
Fat-free mass (kg) [median (range)]	44.55 (41.47–53.28)	53.90 (50.55–59.90)	41.70 (39.65–43.05)	< 0.001
Muscle mass (kg) [median (range)]	40.95 (38.02–49.12)	50.10 (46.75–55.50)	38.10 (36.35–39.45)	< 0.001
Muscle mass category [*n* (%)]				< 0.001
Normal	40 (46.51%)	7 (17.95%)	33 (70.21%)	
Insufficient	25 (29.07%)	13 (33.33%)	12 (25.53%)	
Well-developed	21 (24.42%)	19 (48.72%)	2 (4.26%)	
Basal metabolic rate (kcal) [median (range)]	1078.50 (1018.00-1181.50)	1190.00 (1147.00-1300.50)	1027.00 (985.00-1052.00)	< 0.001
Visceral fat area (cm²) [mean ± SD]	104.42 ± 38.96	125.95 ± 34.48	86.55 ± 33.22	< 0.001
Visceral fat category [*n* (%)]				< 0.001
Subcutaneous	3(3.49%)	2(5.13%)	1(2.13%)	
Balanced	11(12.79)	0(0)	11(23.4%)	
Borderline	13(15.12%)	3(7.69%)	10(21.28%)	
Visceral obesity	58(67.44%)	34(87.18%)	24(51.06)	
Severe visceral obesity	1(1.16%)	0(0)	1(2.13%)	
Phase angle (°) [median (range)]	7.60 (7.40–7.90)	7.90 (7.80-8.00)	7.50 (7.30–7.50)	< 0.001
Appendicular skeletal muscle mass index (kg/m²) [median (range)]	7.82 (7.40–8.59)	8.63 (8.05–9.47)	7.43 (7.04–7.77)	< 0.001

According to the normal range of BMI at different ages in Chinese Residents’ Dietary Guidelines (2022 Edition), middle-aged and elderly people (60–64 years old) 18.5 kg/m^2^-23.9 kg/m^2^, general elderly people (65–79 years old) 20 kg/m^2^-26.9 kg/m^2^, and elderly people (≥ 80 years old) 22 kg/m^2^-26.9 kg/m^2^

### Sarcopenia

According to the 2024 Chinese Guideline for the Diagnosis and Treatment of Sarcopenia, none of the participants met the diagnostic criteria for sarcopenia, as all individuals had muscle mass above the cutoff. However, 12 participants (13.9%) demonstrated lower handgrip strength and were therefore classified as having possible sarcopenia. Compared with participants with normal muscle strength, those with possible sarcopenia were older, although the difference did not reach statistical significance (71.00 vs. 69.00 years, *P* = 0.062). Sex distribution was similar between the possible sarcopenia and normal muscle strength groups. No significant differences were observed in BMI, body fat percentage, fat mass, fat-free mass, muscle mass, basal metabolic rate, visceral fat area, PA, or ASMI (all *P* > 0.05), indicating comparable overall body composition profiles. The clearest distinction between groups was muscle strength: participants with possible sarcopenia had substantially lower handgrip strength (21.64 vs. 30.04 kg, adjusted *P* < 0.001 after controlling for age and sex). Calf circumference and gait speed did not differ significantly between the possible sarcopenia and normal muscle strength groups. Overall, these findings indicate that while sarcopenia was not present in this cohort, early functional decline—manifested primarily as lower muscle strength was detectable among a subset of participants (Table 3).

**Table 3:** Comparison by possible sarcopenia status

Variables	Normal muscle strength (*n* = 74)	Possible sarcopenia (*n* = 12)	*P*-value	Adjusted *P*-value
BMI (kg/m²)	24.077 (23.376–24.779)	24.059 (22.287–25.832)	0.9851	1.0000
Body fat percentage (%)	27.217 (26.016–28.419)	28.447 (25.413–31.480)	0.4577	0.9154
Fat mass (kg)	18.217 (16.983–19.451)	18.343 (15.226–21.459)	0.9409	1.0000
Fat-free mass (kg)	48.411 (47.251–49.571)	45.848 (42.920-48.777)	0.1110	0.2220
Muscle mass (kg)	44.596 (43.544–45.648)	42.176 (39.519–44.833)	0.0973	0.1946
Basal metabolic rate (kcal)	1128.451 (1109.796-1147.107)	1089.497 (1042.385-1136.608)	0.1317	0.2635
Visceral fat area (cm²)	104.360 (96.630-112.090)	118.138 (98.617-137.659)	0.1974	0.3948
Phase angle (°)	7.718 (7.642–7.793)	7.604 (7.414–7.795)	0.2762	0.8287
ASMI (kg/m²)	8.102 (7.944–8.261)	7.846 (7.445–8.246)	0.2423	0.4845
Handgrip strength (kg)	30.042 (29.062–31.021)	21.638 (19.164–24.112)	0.0000	0.0000
Calf circumference (cm)	34.892 (34.226–35.559)	33.562 (31.880-35.244)	0.1492	0.2984
Gait speed (m/s)	1.347 (1.297–1.398)	1.316 (1.189–1.444)	0.6575	1.0000

*P*-values were obtained from analysis of covariance adjusted for age and sex. Adjusted *P*-values were calculated for multiple comparisons using the Holm–Bonferroni method

### Nutritional status

According to the MNA-SF, seven participants were identified as being at risk of malnutrition, while the remaining participants had normal nutritional status. Age distribution and sex composition did not differ significantly between the malnutrition-risk and normal nutritional status groups. Participants at nutritional risk showed significantly poorer body composition indicators. After covariate adjustment and correction for multiple comparisons, they had significantly lower BMI (20.12 vs. 24.42 kg/m², adjusted *P* = 0.0005), lower body fat percentage (22.01% vs. 27.86%, adjusted *P* = 0.010), and lower fat mass (12.22 vs. 18.77 kg, adjusted *P* = 0.004). Visceral fat area was also markedly lower in the malnutrition-risk group (75.20 vs. 109.01 cm², adjusted *P* = 0.028). Markers of muscle status were generally reduced among those at nutritional risk. Fat-free mass and muscle mass were significantly lower (adjusted *P* = 0.019 and *P* = 0.024, respectively), and ASMI was significantly lower (7.18 vs. 8.15 kg/m², adjusted *P* = 0.0007). Calf circumference was also significantly smaller (31.78 vs. 34.97 cm, adjusted *P* = 0.014). No significant differences were observed in PA, handgrip strength, or gait speed (Table 4).

**Table 4:** Comparison by nutritional risk

Variables	Normal nutritional status (*n* = 79)	At risk of malnutrition (*n* = 7)	*P*-value	Adjusted *P*-value
BMI (kg/m²)	24.424 (23.802–25.045)	20.124 (18.039–22.210)	0.0002	0.0005
Body fat percentage (%)	27.863 (26.759–28.966)	22.010 (18.306–25.714)	0.0034	0.0103
Fat mass (kg)	18.765 (17.647–19.883)	12.222 (8.470-15.974)	0.0013	0.0040
Fat-free mass (kg)	48.490 (47.404–49.576)	43.131 (39.487–46.775)	0.0063	0.0188
Muscle mass (kg)	44.643 (43.654–45.632)	39.921 (36.602–43.241)	0.0081	0.0244
Basal metabolic rate (kcal)	1127.874 (1110.004-1145.745)	1068.288 (1008.315-1128.262)	0.0616	0.1848
Visceral fat area (cm²)	109.014 (101.782-116.246)	75.203 (50.933–99.474)	0.0095	0.0284
Phase angle (°)	7.709 (7.636–7.783)	7.620 (7.374–7.865)	0.4881	0.8287
ASMI (kg/m²)	8.145 (8.003–8.287)	7.182 (6.705–7.658)	0.0002	0.0007
Handgrip strength (kg)	28.856 (27.707–30.005)	29.095 (25.240-32.951)	0.9059	0.9059
Calf circumference (cm)	34.966 (34.346–35.586)	31.780 (29.700-33.860)	0.0045	0.0135
Gait speed (m/s)	1.341 (1.292–1.389)	1.369 (1.206–1.533)	0.7364	1.0000

*P*-values were obtained from analysis of covariance adjusted for age and sex. Adjusted *P*-values were calculated for multiple comparisons using the Holm–Bonferroni method

### Frailty screening

According to the FRAIL scale assessment, 39 participants were classified as robust, and 47 participants were classified as prefrail. Age tended to be higher in the prefrail group, although the difference did not reach statistical significance (*P* = 0.056). Sex distribution and BMI were similar between the robust and prefrail groups. Body composition indicators, including fat mass, fat-free mass, muscle mass, and visceral fat area, showed no significant group differences (all *P* > 0.05). Body fat percentage was slightly higher in the prefrail group, but not significantly (*P* = 0.064). After covariate adjustment and correction for multiple comparisons, the prefrail group had significantly lower handgrip strength (27.47 vs. 30.54 kg, adjusted *P* = 0.012), while PA no longer showed a significant difference (adjusted *P* = 0.829). Importantly, gait speed was significantly lower among prefrail participants (1.25 vs. 1.45 m/s, adjusted *P* < 0.001). Calf circumference and ASMI did not differ meaningfully between the robust and prefrail groups (Table 5).

**Table 5:** Comparison of participants by frailty status

Variables	Robust (*n* = 39)	Prefrail (*n* = 47)	*P*-value	Adjusted *P*-value
BMI (kg/m²)	23.990 (23.019–24.961)	24.147 (23.255–25.038)	0.8162	1.0000
Body fat percentage (%)	26.995 (25.331–28.658)	27.721 (26.194–29.248)	0.5284	0.9154
Fat mass (kg)	18.209 (16.501–19.916)	18.256 (16.689–19.824)	0.9678	1.0000
Fat-free mass (kg)	48.643 (47.022–50.263)	47.559 (46.071–49.046)	0.3350	0.3350
Muscle mass (kg)	44.808 (43.337–46.280)	43.796 (42.446–45.147)	0.3216	0.3216
Basal metabolic rate (kcal)	1131.295 (1105.235-1157.355)	1116.064 (1092.142-1139.987)	0.3993	0.3993
Visceral fat area (cm²)	103.782 (93.002-114.562)	108.380 (98.484-118.275)	0.5380	0.5380
Phase angle (°)	7.740 (7.635–7.845)	7.670 (7.574–7.766)	0.3364	0.8287
ASMI (kg/m²)	8.109 (7.888–8.330)	8.031 (7.828–8.234)	0.6109	0.6109
Handgrip strength (kg)	30.540 (28.968–32.112)	27.467 (26.024–28.910)	0.0058	0.0116
Calf circumference (cm)	34.914 (33.982–35.845)	34.534 (33.678–35.389)	0.5559	0.5559
Gait speed (m/s)	1.450 (1.388–1.512)	1.252 (1.195–1.309)	< 0.0001	< 0.0001

*P*-values were obtained from analysis of covariance adjusted for age and sex. Adjusted *P*-values were calculated for multiple comparisons using the Holm–Bonferroni method

Furthermore, multivariable logistic regression analysis demonstrated that higher gait speed (each increment of 0.1 m/s, OR = 0.549, 95% CI: 0.380–0.745, *P* = 0.0004) and greater handgrip strength (OR = 0.887, 95% CI: 0.785–0.984, *P* = 0.0325) were independently associated with a lower likelihood of prefrailty (Table 6).

**Table 6:** Multivariable logistic regression analysis of factors associated with prefrailty

Variables	β	SE	Wald	*P*-value	OR (95% CI)
Age (years)	-0.0098	0.0436	0.0505	0.8223	0.990 (0.908–1.079)
Sex (Male)	0.9807	0.9042	1.1762	0.2781	2.666 (0.479–17.556)
Gait speed (0.1 m/s)	-0.5996	0.1703	12.3917	0.0004	0.549 (0.380–0.745)
Handgrip strength (kg)	-0.1204	0.0563	4.5719	0.0325	0.887 (0.785–0.984)

## Discussion

This study examined body composition, muscle health, physical function, nutritional status, and frailty among northern China migrant elders who overwinter in Hainan. Overall, no elders met the diagnostic criteria for sarcopenia, and most maintained normal nutritional status and engaged in regular physical activity. However, several concerning patterns emerged. A high proportion exhibited excessive body fat, visceral obesity, or early manifestations of muscle weakness and frailty. Muscle strength and gait performance varied across subgroups, with prefrail participants showing significantly lower grip strength and PA, while those at nutritional risk demonstrated poorer body composition profiles. These findings suggest that although this cohort demonstrates active lifestyle patterns, important risks related to muscle quality, adiposity, and early-stage frailty remain.

Rather than implying that seasonal migration directly causes the observed physiological patterns, these findings profile the phenotypic characteristics of winter-relocating older adults. A key finding is the coexistence of normal or even high levels of physical activity with unfavorable body composition patterns. Although 84.9% of participants reported regular walking and overall daily exercise averaged 1.33 ± 0.64 h, more than two-thirds exceeded recommended body fat thresholds, and 68.6% met the criteria for visceral obesity. The predominance of aerobic activities, such as walking, tai chi, calisthenics, and dancing, aligns with previous reports on exercise preferences among older Chinese adults [25, 26], but may not provide sufficient stimulus to preserve muscle mass or strength with advancing age. This is supported by the observation that 33.3% of men and 25.5% of women demonstrated guideline-defined low muscle mass (per the AWGS 2019 criteria), and nearly 14% showed lower handgrip strength—the earliest and most sensitive indicator of lower muscle function [27]. Although ASMI and PA values fell within normal ranges, their downward trend among the 65–79 age group suggests the beginning of age-related muscle deterioration [28, 29].

The high prevalence of visceral obesity (68.6%) represents another critical finding. Because inflammatory biomarkers, dietary intake, and muscle metabolism were not assessed in the present study, mechanistic explanations remain speculative. However, based on existing literature, it is hypothesized that visceral adiposity acts not merely as excess fat storage but as a metabolically active depot producing pro-inflammatory cytokines and adipokines, which may accelerate muscle catabolism and impair muscle protein synthesis [30, 31]. This proposed inflammatory burden might theoretically explain why, despite engaging in daily physical activity, many elders exhibited early signs of muscle weakness. The coexistence of increased adiposity and lower muscle function suggests a potential early trajectory toward sarcopenic obesity; nevertheless, this relationship remains a hypothesis that requires confirmation through future studies incorporating biological markers [32]. Regarding nutritional status, a distinct pattern emerged: although participants at risk of malnutrition exhibited significantly lower muscle and fat mass, their physical performance and handgrip strength remained paradoxically preserved compared to those with normal nutritional status. While only 8.1% were classified as being at risk of malnutrition, those individuals displayed significantly lower BMI, fat mass, visceral fat, ASMI, and calf circumference. This heterogeneity within the cohort is important: it suggests that both ends of the nutritional spectrum—overnutrition and undernutrition—are present simultaneously, each contributing to muscle vulnerability but through different mechanisms. It is hypothesized that underweight elders may suffer from insufficient protein intake or chronic disease, whereas overweight elders may experience inflammation-driven muscle degradation despite adequate or excessive caloric intake [33]. This dual risk reinforces the need for individualized nutritional screening and tailored intervention strategies.

Frailty assessments further revealed that more than half of the cohort were in prefrailty, despite being physically active. This discrepancy underscores an important point: not all physical activity is equivalent in its protective effect. The overwhelming majority of participants engaged in low-intensity aerobic activity, particularly walking. While this supports cardiovascular health, predominance of light aerobic activity without structured resistance training may warrant further investigation in future studies regarding its ability to counteract age-related weakness in muscle strength [34]. The absence of such training likely contributes to the observed vulnerability. Furthermore, it is hypothesized that frailty in this cohort may be associated with additional factors encountered during seasonal migration, including changes in dietary patterns, alterations in original life routines, unfamiliarity with local medical resources, and limited access to structured exercise facilities [35]. The combined pattern—normal muscle mass but lower function, high adiposity but normal nutritional screening, and active lifestyle but significant early -stage prefrailty—leads us to hypothesize that this cohort demonstrates early vulnerability despite preserved structural indices. This reflects a discrepancy where preserved muscle mass does not guarantee functional strength, aligning with ongoing scientific debates regarding the limitations and inconsistencies of relying solely on muscle mass assessments [36]. This early vulnerability may reflect a mismatch between lifestyle behaviors (dominated by light aerobic exercise), physiological demands of aging (greater need for strength training and high-quality protein), and environmental/contextual factors (seasonal relocation and changing support systems). Recognizing this mismatch is crucial for optimizing health interventions.

Overall, these findings underscore the need for reinforced interventions aimed at preserving muscle mass, reducing visceral fat accumulation, and preventing frailty progression. Evidence supports the incorporation of resistance-based training as the most effective strategy for improving muscle strength and physical performance [37]. Additionally, dietary adjustments emphasizing adequate protein intake (≥ 1.2 g/kg/day) and appropriate vitamin D supplementation may further benefit muscle health and functional outcomes [38, 39]. Establishing simple, routine screening for sarcopenia and frailty, using indicators such as handgrip strength and calf circumference, may also facilitate early identification and intervention among migrant elders.

This study has several limitations. First, as an inherent limitation, the cross-sectional design explicitly precludes any causal inferences; the observed results reflect descriptive associations rather than temporal changes. Second, no pre-migration baseline data were available, restricting comparisons of lifestyle changes before and after seasonal relocation. Third, body composition was assessed using BIA rather than DXA. While BIA is practical, it is less accurate for precise muscle mass quantification. Furthermore, the zero prevalence of confirmed sarcopenia in this study warrants critical interpretation. Several factors likely contribute to this observation. First, the relatively young age distribution of the sample and their active lifestyle likely protect against severe muscle loss. Second, the reliance on WeChat for recruitment inherently introduces a healthy volunteer bias, attracting older adults with better mobility, thereby excluding those who are frailer. Third, technical differences between BIA and DXA could influence muscle mass estimation. Finally, the applicability of the AWGS 2019 cut-offs to this specific northern seasonal migrant population remains uncertain, as these thresholds were derived from general community-dwelling Asian populations and might not capture early-stage functional declines specific to mobile elders. Fourth, physical activity was self-reported, lacking objective measures of exercise intensity, and precise resistance training data were not captured. Furthermore, detailed dietary intake data (especially regarding precise protein consumption) were not collected, restricting a comprehensive analysis of nutritional influences. Fifth, the sample size was relatively small (*n* = 86), which limits the statistical power for subgroup analyses (e.g., only 7 participants were at nutritional risk). Finally, a potential sampling bias exists, as volunteers recruited via the hospital’s WeChat public account typically have better mobility and overall health status. As highlighted by recent literature on online-based recruitment for older adults, digital strategies often encounter challenges related to the digital divide and limited digital literacy, frequently resulting in limited sociodemographic diversity within recruited samples [40]. Consequently, our WeChat-based recruitment likely attracted a specific subset of older adults with higher e-health literacy and better cognitive and physical functioning. This selection bias inherently limits the generalizability of our findings and likely leads to a significant underestimation of the true prevalence of possible sarcopenia and frailty in the broader winter-relocating older adult population. Additionally, the generalizability of these findings is restricted. Because the sample exclusively comprises northern Chinese older adults migrating to Hainan, the observed patterns may not be directly applicable to non-migrant populations, older adults relocating to different geographical environments, or seasonal migrants in other cultural contexts.

## Conclusions

In summary, this study provides initial evidence that northern migrant elders generally maintain active lifestyles and normal nutritional status, yet exhibit high rates of visceral obesity and multiple early markers of muscle deterioration and frailty. Although sarcopenia was not diagnosed, the winter-relocating older adults demonstrated preserved muscle mass but early functional vulnerability and elevated adiposity, underscoring the need for multidimensional screening approaches beyond muscle mass alone. Given these findings, early monitoring and regular frailty screening are recommended. Furthermore, our findings carry potential implications for future preventive strategies, suggesting a role for structured resistance training and nutritional optimization, although their efficacy requires further evaluation. Further longitudinal research is needed to clarify how seasonal migration influences long-term muscle health and functional outcomes in this uniquely mobile and growing population.

## Supplementary Information

Supplementary Material 1.

## Data Availability

All data generated or analysed during this study are included in this published article.

## Abbreviations

BMI: Body Mass Index
BIA: Bioelectrical Impedance Analysis
DXA: Dual-energy X-ray Absorptiometry
PA: Phase Angle
MNA-SF: Mini Nutritional Assessment-Short Form
ASMI: Appendicular Skeletal Muscle Index
AWGS: Asian Working Group for Sarcopenia
SMI: Skeletal Muscle Mass Index
ANOVA: Analysis of Variance
SD: Standard Deviation
ANCOVA: Analysis of Covariance
OR: Odds Ratio
CI: Confidence Interval
