# Supplementary material for: Body composition, sarcopenia, and frailty among Northern seasonal migrant older adults: a cross-sectional study
Source: BMC Public Health. 2026 Aug 8;26:3209. doi: 10.1186/s12889-026-28960-3 (PMC13737620; doi:10.1186/s12889-026-28960-3)
Supplement: Supplementary file 1 [file 12889_2026_28960_MOESM1_ESM.docx]

**Table S1. Characteristics of body composition analysis of the migrant elder with different ages**

|  | **Overall**  **(n=86)** | **60-64 year**  **(n=17)** | **65-79 year**  **(n=64)** | **≥80 year**  **(n=5)** | **P** |
| --- | --- | --- | --- | --- | --- |
| **Age (years) [median (range)]** | 70.00 (65.25-74.00) | 61.00 (60.00-62.00) | 70.00 (68.00-74.25) | 82.00 (81.00-82.00) | <0.001 |
| **Sex [n (%)]** |  |  |  |  | 0.581 |
| Male | 39 (45.35%) | 9 (52.94%) | 27 (42.19%) | 3 (60.00%) |  |
| Female | 47 (54.65%) | 8 (47.06%) | 37 (57.81%) | 2 (40.00%) |  |
| **BMI (kg/m^2^) [mean±SD]** | 24.05±2.99 | 23.89±2.88 | 23.96±3.08 | 25.78±1.81 | 0.417 |
| **BMI category [n (%)]** |  |  |  |  | <0.001 |
| Below standard | 7 (8.14%) | 0 (0.00%) | 7 (10.94%) | 0 (0.00%) |  |
| Normal | 16 (18.60%) | 12 (70.59%) | 0 (0.00%) | 4 (80.00%) |  |
| Above standard | 63 (73.26%) | 5 (29.41%) | 57 (89.06%) | 1 (20.00%) |  |
| **Body fat percentage (%) [median (range)]** | 29.00 (24.22-32.18) | 27.90 (23.10-29.90) | 29.15 (24.23-32.85) | 29.80 (24.60-34.40) | 0.485 |
| **Body fat percentage category [n (%)]** |  |  |  |  | 0.276 |
| < Standard | 22 (25.58%) | 6 (35.29%) | 16 (25.00%) | 0 (0.00%) |  |
| ≥ Standard | 64 (74.42%) | 11 (64.71%) | 48 (75.00%) | 5 (100.00%) |  |
| **Fat mass (kg) [median (range)]** | 18.35±5.42 | 17.70±5.56 | 18.22±5.45 | 22.36±3.24 | 0.223 |
| **Fat-free mass (kg) [median (range)]** | 44.55 (41.47-53.28) | 47.90 (41.90-55.80) | 44.20 (41.08-50.95) | 61.90 (41.80-62.10) | 0.278 |
| **Muscle mass (kg) [median (range)]** | 40.95 (38.02-49.12) | 44.30 (38.40-51.30) | 40.65 (37.77-47.25) | 57.20 (38.20-57.40) | 0.263 |
| **Basal metabolic rate (kcal) [median (range)]** | 1078.50 (1018.00-1181.50) | 1143.00 (1076.00-1300.00) | 1052.00 (1002.75-1157.00) | 1265.00 (973.00-1277.00) | 0.020 |
| **Visceral fat area (cm²) [mean±SD]** | 104.42±38.96 | 98.00±45.32 | 103.94±37.30 | 132.40±31.23 | 0.219 |
| **Phase angle (°) [median (range)]** | 7.60 (7.40-7.90) | 7.70 (7.50-7.90) | 7.60 (7.40-7.90) | 7.70 (7.40-7.90) | 0.822 |
| **Appendicular skeletal muscle mass index (kg/m²) [median (range)]** | 7.82 (7.40-8.59) | 8.06 (7.26-8.74) | 7.78 (7.42-8.37) | 9.08 (7.51-9.15) | 0.573 |
